# Supplementary material for: Effect of collagen casing on the quality characteristics of fermented sausage
Source: PLoS One. 2022 Feb 3;17(2):e0263389. doi: 10.1371/journal.pone.0263389 (PMC8812867; doi:10.1371/journal.pone.0263389)
Supplement: Attachment — Submitted filename: Response to editor.docx [file pone.0263389.s009.docx]

1. Thank you for updating your data availability statement. You note that your data are available within the Supporting Information files, but no such files have been included with your submission. At this time we ask that you please upload your minimal data set as a Supporting Information file, or to a public repository such as Figshare or Dryad.
Please also ensure that when you upload your file you include separate captions for your supplementary files at the end of your manuscript.
As soon as you confirm the location of the data underlying your findings, we will be able to proceed with the review of your submission.
**Response to editor:** Sorry for this fault, we have uploaded our study’s minimal underlying data set as Supporting Information file this time.
2. We note that the grant information you provided in the ‘Funding Information’ and ‘Financial Disclosure’ sections do not match.
When you resubmit, please ensure that you provide the correct grant numbers for the awards you received for your study in the ‘Funding Information’ section.

**Response to editor:** We have deleted “Funding Information” part in our revised manuscript and have ticked the box for ‘The author(s) received no specific funding for this work” in my system.
