# Supplementary material for: Effect of collagen casing on the quality characteristics of fermented sausage
Source: PLoS One. 2022 Feb 3;17(2):e0263389. doi: 10.1371/journal.pone.0263389 (PMC8812867; doi:10.1371/journal.pone.0263389)
Supplement: Attachment — Submitted filename: 2nd Response to Reviewers.docx [file pone.0263389.s010.docx]

Dear editor:

Thank you for providing these comments on our submission entitled “Effect of Collagen Casing on the Quality Characteristics of Fermented Sausage”. We appreciate the constructive comments from you and five reviewers. We have studied the comments carefully and provided a detailed response to each point raised.

**Response to Reviewer**

***Question 1:*** The P letter for statistical value should be an uppercase-italic face letter. Amend throughout the text.

***Response to question 1:*** Thanks for your careful review. We have corrected the mistake in our revised manuscript (line 35, 36, 38, page 2).

***Question*** *2****:*** Line 33: The results showed that with storage time increasing...amend to The results showed that with increasing the storage time.

***Response to question 2:*** Thanks for your suggestion. We have corrected relevant content in our revised manuscript (lines 31, page 2).

*Question 3:* Lines 81-91: Raise a question about the novel aspect of the current study. If there were several studies (2,5, 13, 14, 15), then "what does the current MS add to the existing knowledge"? This Must be discussed and interpreted in the introduction.

***Response to question 3:*** Thanks for your suggestion. We have added relevant content in our revised manuscript (lines 95-96, pages 5-6).

***Question 4:*** Full vendor details should include company, city (state) and country. Amend throughout the text.

***Response to question 4:*** Sorry for this mistake. We have corrected the mistake in our revised manuscript (line 105-108, 118, 120, 123, 137, 148-149, 155, 161,163, 165,182 pages 6-9).

***Question 5:*** Use mL, uL, L, dL throughout the MS.

***Response to question 5:*** Sorry for this mistake. We have corrected the mistake in our revised manuscript (line 150 page 8).

***Question 6:*** Check spaces and punctuation throughout the MS.

***Response to question 6:*** Thank you for your constructive suggestion. We have added relevant content in our revised manuscript (lines 105, 159, pages 6, 8).

***Question 7:*** Section: Determination of biogenic amines: You should provide the details of method performance. This should include calibration curves, linearity, LOD, LOQ, accuracy and precision of quantified biogenic amine.

***Response to question 7:*** Thank you for your constructive suggestion. We have added relevant content in our revised manuscript (lines 191-195, page 10).

***Question 8:*** Data in Tables should be presented as mean +/-SD, not SE.

***Response to question 8:*** Sorry for this funny mistake. Actually we have presented our data as mean±SD. We have modified mean±SE to mean±SD in our revised manuscript (lines 209, 224, 237, 243, 257, 274 pages 11-14, 16). If you have any doubts, please check our original data uploaded before.

***Question 9:*** Conclusion: Rewrite it again as it repeats what has been stated in the abstract.

***Response to question 9:*** Thank you for your suggestion. We have added relevant content in our revised manuscript (lines 335-337, page 19).
