# Supplementary material for: Effect of collagen casing on the quality characteristics of fermented sausage
Source: PLoS One. 2022 Feb 3;17(2):e0263389. doi: 10.1371/journal.pone.0263389 (PMC8812867; doi:10.1371/journal.pone.0263389)
Supplement: Attachment — Submitted filename: Response to Reviewers.docx [file pone.0263389.s011.docx]

Dear editor:

Thank you for providing these comments on our submission entitled “Effect of Collagen Casing on the Quality Characteristics of Fermented Sausage”. We appreciate the constructive comments from you and five reviewers. We have studied the comments carefully and provided a detailed response to each point raised.

**Response to Reviewer 2**

***Question 1:*** The manuscript must describe a technically sound piece of scientific research with data that supports the conclusions. Experiments must have been conducted rigorously, with appropriate controls, replication, and sample sizes. The conclusions must be drawn appropriately based on the data presented.

***Response to question 1:*** We have checked our experiment design and original data, meanwhile have added some experiments on volatile components to make our results experiments more complete.

***Question*** *2****:*** Although the authors improved the quality of the manuscript, they did not incorporate the additional data recommended during the past reviews and the manuscript is still superficial. The authors were advised to add data related to the variations of fats, fatty acid, and other common volatile components, but failed to do so claiming they will consider such results for another publication. Besides, results from optical microscope (SEM) analysis are still lacking. With a focus on some biogenic amines and a few physical characteristics only, the manuscript is not in the required standard to be considered for publication.

***Response to question 2:*** Thank you for your suggestion. we have measured the fermented sausage flavor and showed relevant contents in our revised manuscript (line 36-37, page 2, line 180-184, page 9, 269-286, 15-18, 336-349, page 21-22 and table 6). Our research mainly focused on the effect of different casing types on the quality characteristics of fermented sausage. The research on the organizational structure of casings itself was not the focus of this research. Your valuable suggestions have been involved in our subsequent research. Please keep an eye on our research progress.

**Response to Reviewer 3**

***Question 1:*** The authors have incorporated the comments. I understand that running extra texture measurements and incorporate permeability measurements is not possible. Maybe a suggestion for future articles. This article is now ready for publishing.

***Response to question 1:*** Thank you very much for your valuable suggestions. We will adopt the measurement of relevant indicators you proposed in the following research. Please keep paying attention to us.
