# Supplementary material for: Effect of collagen casing on the quality characteristics of fermented sausage
Source: PLoS One. 2022 Feb 3;17(2):e0263389. doi: 10.1371/journal.pone.0263389 (PMC8812867; doi:10.1371/journal.pone.0263389)
Supplement: Attachment — Submitted filename: response to reviewers.docx [file pone.0263389.s012.docx]

Dear editor:

Thank you for providing these comments on our submission entitled “Effect of Collagen Casing on the Quality Characteristics of Fermented Sausage”. We appreciate the constructive comments from you and five reviewers. We have studied the comments carefully and provided a detailed response to each point raised.

**Response to Reviewer 2**

***Question 1:*** ThIs the manuscript technically sound, and do the data support the conclusions? The manuscript must describe a technically sound piece of scientific research with data that supports the conclusions. Experiments must have been conducted rigorously, with appropriate controls, replication, and sample sizes. The conclusions must be drawn appropriately based on the data presented.

***Response to question 1:*** We have checked our experiment design and original data, meanwhile have added some experiments on volatile components to make our results experiments more complete.

***Question*** *2****:*** Is the manuscript presented in an intelligible fashion and written in standard English?

PLOS ONE does not copyedit accepted manuscripts, so the language in submitted articles must be clear, correct, and unambiguous. Any typographical or grammatical errors should be corrected at revision, so please note any specific errors here.

***Response to question 2:*** Thanks for your careful check. We sought for the help from AJE (American Journal Experts) to refine our manuscript ([D3C61K32](https://secure.aje.com/cn/researcher/index/details/D3C61K32)).

***Response to question 2:***

***Question 3:*** With the addition of data related to volatile components, the content of the manuscript has now improved to some extent. Related to this, the authors should clarify the method regarding the analysis of volatile components (lines 180-184). Specifically, the modified method, the identification procedure, and the quantification techniques along with key GC-MS parameters should be described. More importantly, it would be great if the authors incorporate the effect of storage time (21 d, 41 d and 61d) on the variations of these volatile molecules (as they did for biogenic amines).

***Response to question 3:*** Thank you for your suggestion. We have added relevant contents such as the determination method, results and discussion of fermented sausage flavor. (line36-37, 48, 185-189, 273-285, 374-375, page 2, 3, 9-10, 15-20, 25).
